# Risk factor-based optimal endoscopic surveillance intervals after endoscopic submucosal dissection for gastric adenoma

**DOI:** 10.1038/s41598-021-00969-1

**Published:** 2021-11-01

**Authors:** Choong-Kyun Noh, Eunyoung Lee, Gil Ho Lee, Sun Gyo Lim, Kee Myung Lee, Jin Roh, Young Bae Kim, Bumhee Park, Sung Jae Shin

**Affiliations:** 1 Department of Gastroenterology, Ajou University School of Medicine, 164, Worldcup-ro, Yeongtong-gu, Suwon, Gyeonggi-do 16499 Republic of Korea; 2 Department of Biomedical Informatics, Ajou University School of Medicine, 164, Worldcup-ro, Yeongtong-gu, Suwon, Gyeonggi-do 16499 Republic of Korea; 3 Office of Biostatistics, Ajou Research Institute for Innovative Medicine, Ajou University Medical Center, Suwon, Republic of Korea; 4 Department of Medical Sciences, Biomedical Informatics, Graduate School of Ajou University, Suwon, Republic of Korea; 5 Department of Pathology, Ajou University School of Medicine, Suwon, Republic of Korea

**Keywords:** Risk factors, Stomach diseases, Gastroenterology, Oesophagogastroscopy

## Abstract

To date, there exists no established endoscopic surveillance interval strategy after endoscopic submucosal dissection (ESD) for gastric adenoma. In this study, we suggest a risk factor-based statistical model for optimal surveillance intervals for gastric adenoma after ESD with curative resection. A cox proportional hazard model was applied to identify risk factors for recurrence after ESD. Patients (n = 698) were categorized into groups based on the identified risk factors. The cumulative density of recurrence over time was computed using a cubic splined baseline hazard function, and the customized surveillance interval was modeled for each risk group. The overall cumulative incidence of recurrence was 7.3% (n = 51). Risk factors associated with recurrence were male (hazard ratio [HR], 2.60, *P* = 0.030), protruded scar (HR, 3.18, *P* < 0.001), and age ≥ 59 years (HR, 1.05, *P* < 0.001). The surveillance interval for each group was developed by using the recurrence limit for the generated risk groups. According to the developed schedule, high-risk patients would have a maximum of seven surveillance visits for 5 years, whereas low-risk patients would have biennial surveillance for cancer screening. We proposed a simple and promising strategy for determining a better endoscopic surveillance interval by parameterizing diverse and group-specific recurrence risk factors into a well-known survival model.

## Introduction

Gastric adenoma is recognized as a premalignant lesion since it can develop into gastric cancer^1–3^. The diagnosis of gastric adenoma is made after biopsy during the endoscopic evaluation. However, due to the discrepancy between the pathologic results of the endoscopic forceps biopsy and the resected specimen, the final diagnosis can be upgraded to cancer^4–6^. In particular, adenoma with high-grade dysplasia showed higher rates of discrepancy after upgradation^6^. Therefore, when gastric adenoma is detected through endoscopic examination, it needs to be removed, and the complete histopathologic work-up should be performed^7^.

Endoscopic submucosal dissection (ESD) is a minimally invasive technique to remove superficial gastric neoplasms. Since it enables both en-bloc resection and precise histopathologic evaluation, ESD may determine whether curative resection was done^8^. However, in studies which evaluated long-term outcomes, the recurrence rate of up to 20.3% was reported after removal of gastric adenomas with endoscopic resection^9^. Thus, endoscopic surveillance is necessary after adenoma removal with ESD. However, there is no consensus on surveillance when gastric adenoma is removed by ESD. Especially, there is no exact guideline on the surveillance ‘interval’ in gastric adenoma after ESD. Surveillance is conducted in the same manner as in ealy gastric cancer (EGC)^10^, sometimes even without a follow-up.

In this study, we suggest a risk factor-based statistical model for optimal surveillance intervals for gastric adenoma after ESD with curative resection. We assumed that the recurrence rate would be different between patients with a high risk of recurrence and those with low risk, although potential risk factors for recurrence have not yet been identified. Literature for risk factors of recurrence after ESD with curative resection is either rare or non-existent. Thus, we hypothesized that the surveillance interval could be distributed differently depending on the risk factors for recurrence in patients who had undergone ESD. To this end, we first identified potential risk factors for the recurrence and then proposed surveillance intervals developed by parameterizing the factors identified into the Cox proportional hazard model.

## Methods

### Patients

This retrospective, single-center study was conducted at the Ajou University Medical Center (Suwon, Republic of Korea). In total, 3331 patients underwent ESD for gastric neoplasm at our center between March 2005 and March 2018. The inclusion criteria for this study were as follows: (1) histopathologic confirmation of adenoma; and (2) histopathological curative resection^10^. Exclusion criteria were as follows: (1) perforation (both micro- and macro-) during ESD; (2) total follow-up period of 6 months or less; (3) no endoscopic follow-up at 3 months; and (4) non-diagnosis in the final pathology. The study protocol was approved by the Ajou University Hospital Institutional Review Board (approval no. AJIRB-MDB-18-104), which waived the requirement for individual informed consent owing to the retrospective nature of the study. All methods were carried out in accordance with relevant guidelines and regulations.

### ESD procedures and histopathologic evaluation

Three expert endoscopists (LSG, LKM, and SSJ) performed all ESD procedures, with single-channel endoscopy (GIF-Q260J; Olympus, Tokyo, Japan) or two-channel endoscopy (GIF-2TQ260M; Olympus, Tokyo, Japan). The entire stomach was examined before ESD to check whether there were other lesions. After identifying the lesion by narrow band imaging and chromoendoscopy using indigo carmine, circumferential marking was performed using a needle knife (Dual knife; Olympus, Tokyo, Japan) or through argon plasma coagulation (Erbe Elektromedizin, Tübingen, Germany). The epinephrine-mixed fluid was injected for submucosal lifting, and dissection was performed using an insulated-tip knife (IT knife; Olympus, Tokyo, Japan). All samples were fixed in 10% buffered formalin solution and embedded in paraffin. A standard histopathological process including hematoxylin and eosin staining was conducted. Pathological diagnoses were made according to the revised Vienna classification^11^. Protuberant scars with a polypoid or nodular shape located at the post-ESD site were defined as protruded scars (Supplement Fig. 1)^12^.

### Definition of ESD outcomes and recurrence

*En*-bloc resection was defined as the complete removal of a lesion in one-piece without fragmentation (single final specimen). Resection was defined as curative when specific conditions (*En-*bloc resection, negative horizontal and vertical margins on histologic examination, no lymphovascular infiltration, and no perineural invasion) were met^10^. Residual disease was defined as a recurrence of tumor at the ESD site within one year after ESD^13^. Local recurrence was defined as a recurrence at the ESD site for more than one year after ESD^13^. Distant metastasis was defined as the recurrence or tumor outside the stomach^13^. The synchronous lesion was defined as a new lesion that recurred at a different location from the ESD site in the stomach within one year after ESD^13^. The metachronous lesion was defined as a new lesion that developed more than one year after ESD in the stomach. Since surveillance needs to detect all cases of recurrence, we defined ‘recurrence’ as all adenoma or cancer detected in surveillance endoscopy after curative resection.

### Definitions of candidate risk factors for recurrence

The risk factors for recurrence after ESD of gastric adenoma were sex, age, number of ESD lesion (single, multiple), presence of atrophy, presence of metaplasia, presence of *Helicobacter pylori (H. pylori)* infection, success of *H. pylori* eradication, tumor location (upper 1/3, middle 1/3, lower 1/3), gross morphology (flat/elevated, depressed), lesion diameter (< 10 mm, 11–20 mm, 21–30 mm, > 30 mm), presence of ulceration at initial work up, presence of ulceration at 3 months f/u (first follow up), presence of fibrosis during ESD, pathologic grade of differentiation (low grade adenoma, high grade adenoma), histopathologic discrepancy between endoscopic forceps biopsy and post-ESD pathology (concordant: when the initial diagnosis and ESD specimens were the same, upgraded: when the diagnoses of subsequent ESD specimens showed a histology of more malignant potential [e.g., from low-grade dysplasia to high-grade dysplasia], or down-graded: when the diagnoses of subsequent ESD specimens showed a histology of less malignant potential [e.g., from high-grade dysplasia to low-grade dysplasia]), scar type at the 3 month follow-up (flat of protruded), safety margin space (area of the specimen − area of the lesion), and total procedure time (from the circumferential marking around the lesion to the completion of hemostasis after complete removal). We defined atrophy and metaplasia based on the endoscopic findings. When necessary, pathologic confirmation was performed. Atrophy was defined as visibility of the vascular pattern in the mucosa, while intestinal metaplasia was defined as replacement of the surface, foveolar, and glandular epithelium in the oxyntic or antral mucosa by intestinal epithelium^14^. We defined a protruded scar as a scar formation characterized by a polypoid or nodular shaped protuberant scar located in the post-ESD site (Supplement Fig. 1)^12^. Submucosal fibrosis during ESD occurring following the injection of a solution containing indigo carmine into the submucosal layer was defined as follows: F0, no fibrosis (only observed as a blue transparent layer); F1, mild fibrosis (a white web-like structure); and F2, severe fibrosis (a white muscle-like structure without a blue transparent layer)^15^. We considered only the absence (F0) and presence (F1 and F2) of submucosal fibrosis as potential risk factors in the analysis.

### *Helicobacter pylori* evaluation

The status of *H. pylori* infection was evaluated via a rapid urease test and histologically confirmed via hematoxylin and eosin and Wright-Giemsa staining before ESD. *H. pylori* infection status was considered positive when one or both of these tests were positive. If a patient was infected with *H. pylori*, eradication was performed after ESD, and a follow-up rapid urease test or urea breath test was conducted.

### Follow-up schedule

In our center, follow-up endoscopy was performed at 3, 6, 12, 18, and 24 months after ESD and annually thereafter. At all follow-ups, four biopsy samples were taken from ESD sites. Recurrence was also checked in sites other than the ESD site, and a biopsy was taken at the endoscopist’s discretion.

### Statistical analysis and development of proposed surveillance intervals

For baseline characteristics, continuous variables were compared using the t-test, and categorical variables were compared using the chi-squared test. The surveillance interval was analyzed for each case. Risk factors were identified using a Cox proportional hazard model, and a stepwise selection method was used to select the best subset of predictors among risk factor candidates (alpha-to-enter = 0.25, alpha-to-remove = 0.15)^16^. Identified risk factors were classified into risk groups. If any continuous variables (age, total procedure time, safety margin space) were selected, they were converted to binary variables for easier classification and a quick clinical decision by determining the optimal cut point for a continuous variable in the Cox proportional regression model^17^.

The Cox regression model with interval censoring was applied to compute the cumulative density of recurrence after ESD. Its baseline hazard function was specified with either a cubic spline model or a piecewise-constant model^18^. The parameterization of the baseline hazard function was examined with 2 to 4 degrees of freedom or 2 to 5 equally spaced intervals and selected based on the lowest Akaike Information Criterion and Bayesian Information Criterion. With each classified risk group, the cumulative density of recurrence over time was estimated from fitting the Cox regression model with interval censoring. Its baseline hazard function for recurrence after ESD was smoothened by a cubic spline with 3 and 2 degrees of freedom, respectively. Let $t$ be the time until the recurrence which has occurred within an interval of time such that ${L}_{i}<t\le {R}_{i}$ where ${L}_{i}$ and ${R}_{i}$ is the lower and upper bound of time interval for individual $i$, respectively. The Cumulative density of recurrence can be written as follows:

$$F\left(t\right)=1-S\left(t\right)=1-\mathrm{exp}[-{\Lambda }_{0}\left(t\right)\mathrm{exp}\left({Z}_{i}^{^{\prime}}\beta \right)]$$

where ${\Lambda }_{0}$ is the baseline cumulative hazard function, ${Z}_{i}$ is the vector of explanatory covariates for individual $i$, $\beta$ is the vector of regression coefficients.

Surveillance interval modeling required a set of recurrence limits at the follow-up. The values of the recurrence limit were determined on the basis of the literature^9,19–23^ and our data. The surveillance model developed in this study assumed that there should be no recurrence at the first follow-up (3 months) after ESD. ‘Surveillance intervals’ were selected such that the risk of recurrence at each surveillance visit would be equal or less than the tolerable risk limit, and the risk at each surveillance visit should be approximately equal. Therefore, the proposed surveillance interval would not exceed the recurrence limit at any visit, and the mean recurrence risk per visit was less or equal to the limit. All statistical analyses were two-sided and were performed using SAS statistical software, version 9.4 (SAS Institute, Cary, NC, USA) and *P*-values < 0.05 were considered statistically significant.

## Results

### Baseline characteristics

**Figure 1:**
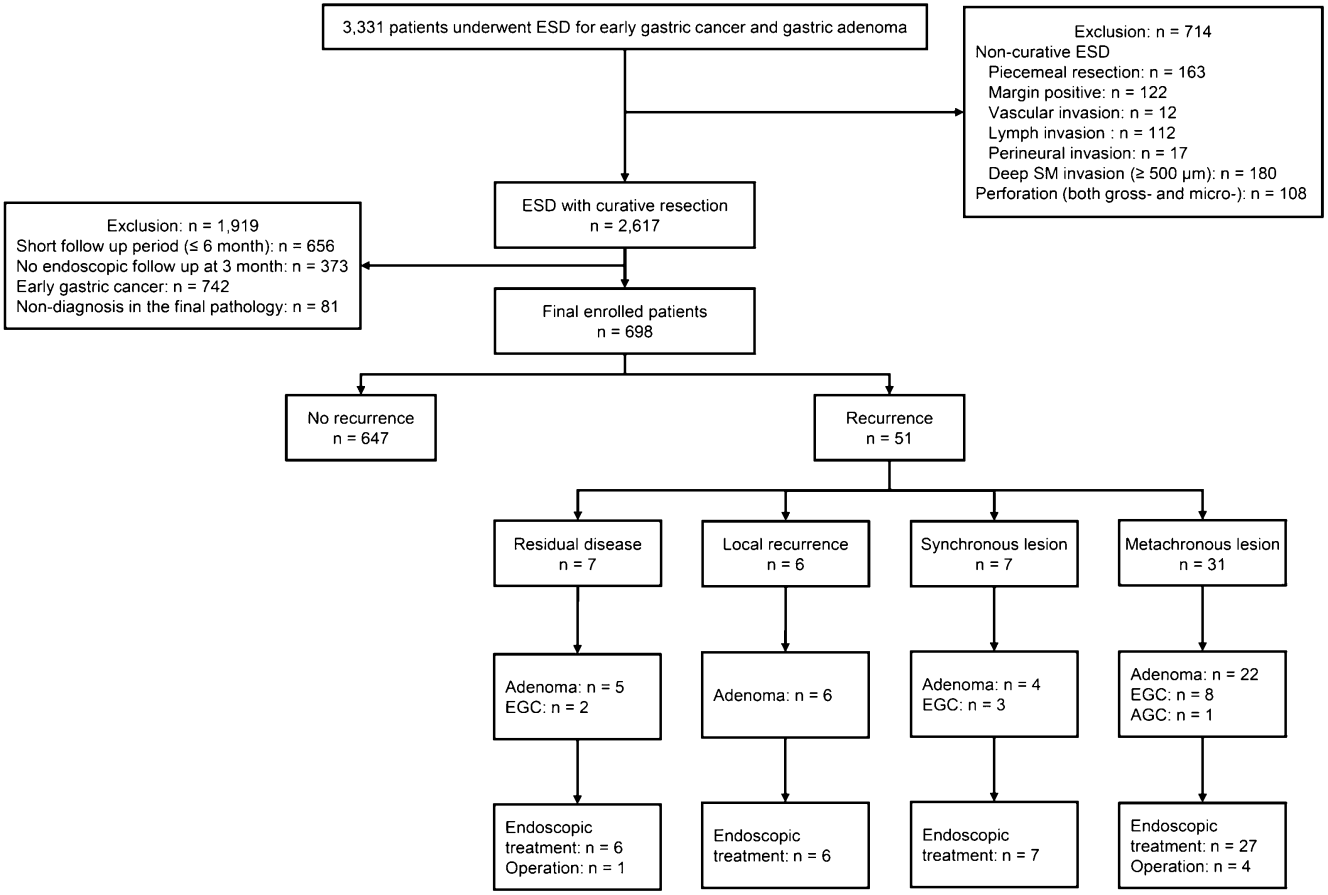
Flow diagram of enrolled patients.

The flow diagram of the enrolled patients is shown in Fig. 1. The incidence was 1.9% for recurrence at the previous ESD site and recurrence in the stomach other than at the ESD site was the 5.4%, respectively. Among the recurrence case, 14 patients (27.5%) had a cancer recurrence (EGC: 13, advanced gastric cancer: 1). The baseline characteristics of patients depending on recurrence are shown in Table 1. There were significant differences in age, sex, atrophy, metaplasia, fibrosis, and scar morphology between the non-recurrence and recurrence groups.

**Table 1:** Baseline characteristics of enrolled patients after endoscopic submucosal dissection with curative resection.

	Total (n = 698)	No recurrence (n = 647)	Recurrence (n = 51)	*P-*value*
Age, mean ± SD, years	61.8 ± 10.0	61.5 ± 10.1	66.0 ± 8.7	< 0.01
**Sex, n (%)**				0.01
Male	498 (71.3)	454 (70.2)	44 (86.3)	
Female	200 (28.7)	193 (29.8)	7 (13.7)	
**Location, n (%)**				0.60
Upper third	28 (4.0)	27 (4.2)	1 (2.0)	
Middle third	256 (36.7)	239 (36.9)	17 (33.3)	
Lower third	414 (59.3)	381 (58.9)	33 (64.7)	
**Lesion diameter**				
Mean ± SD, mm	17.9 ± 8.3	17.8 ± 8.3	19.4 ± 8.5	0.17
≤ 10 mm, n (%)	150 (21.5)	143 (22.1)	7 (13.7)	
11–20 mm, n (%)	365 (52.3)	338 (52.2)	27 (52.9)	
21–30 mm, n (%)	137 (19.6)	125 (19.3)	12 (23.5)	
> 30 mm, n (%)	46 (6.6)	41 (6.3)	5 (9.8)	
**Gross morphology type, n (%)**				0.20
Elevated/flat	669 (95.8)	618 (9.5)	51 (100.0)	
Depressed	29 (4.2)	29 (4.5)		
**Number of lesion, n (%)**				
Single	634 (90.8)	592 (91.5)	42 (82.4)	0.04**
Multiple	64 (9.2)	55 (8.5)	9 (17.6)	
**Atrophy gastritis, n (%)**	500 (71.6)	454 (70.2)	46 (90.2)	< 0.01
Antrum	180 (36.0)	171 (37.7)	9 (19.6)	
Expanded to the lesser curvature of the body	269 (53.8)	240 (52.9)	29 (63.0)	
Entire stomach	51 (10.2)	43 (9.4)	8 (17.4)	
Intestinal metaplasia, n (%)	534 (76.5)	489 (75.6)	45 (88.2)	0.04
***Helicobacter pylori* infection, n (%)**				0.95
Current	203 (29.1)	188 (29.1)	15 (29.4)	
Previous (including eradicated)	167 (23.9)	154 (23.8)	13 (25.5)	
None	328 (47.0)	301 (46.5)	23 (45.1)	
Specimen area − lesion area^a^, mean ± SD, mm^2^	2.4 ± 1.4	2.4 ± 1.3	2.7 ± 1.6	0.19
Total procedure time	50.8 ± 32.7	51.2 ± 33.3	46.2 ± 24.6	0.30
Fibrosis during ESD, n (%)	131 (18.8)	127 (19.6)	4 (7.8)	0.04
Presence of ulceration at 1st follow-up, n (%)	17 (2.4)	16 (2.5)	1 (2.0)	0.82
**Scar morphology at 1st follow-up**^**b**^**, n (%)**				< 0.001
Flat scar	642 (92.0)	602 (93.0)	40 (78.4)	
Protruded scar	56 (8.0)	45 (7.0)	11 (21.6)	
**Discrepancy, n (%)**				0.85
Downgrade and concordant	647 (92.8)	600 (92.9)	47 (92.2)	
Upgrade	50 (7.2)	46 (7.1)	4 (7.8)	

ESD, endoscopic submucosal dissection; n, number; NA, not applicable; SD, standard deviation.

*Non-recurrence vs. recurrence.

**Fisher’s exact test was applied.

^a^(Area of the specimen − area of the lesion)/1000.

^b^If ulceration was observed at the first follow-up, scar morphology was evaluated according to endoscopy at the 6-month follow-up.

### Incidence and pathologic characteristics of recurrent tumor

**Figure 2:**
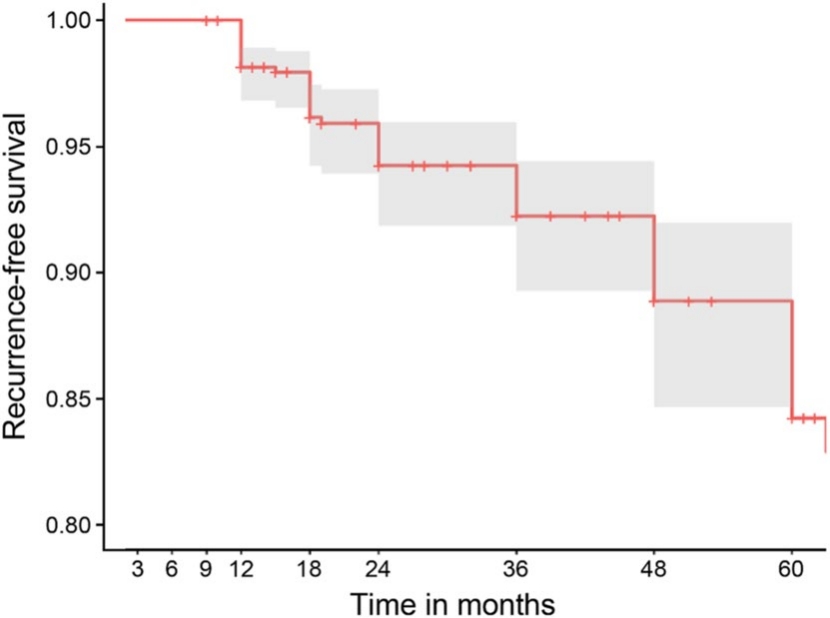
Incidence of recurrence after endoscopic submucosal dissection with curative resection in the Kaplan–Meier plot.

The median follow-up period was 24 months (interquartile range, 12 to 36 months) with a mean of 3.7 ± 1.9 visits per patient. The Kaplan–Meier plot shown in Fig. 2 was used to graphically examine the overall incidence of recurrence. The incidence of total recurrence was 7.3% and the characteristics of recurrent tumor are shown in Table 2.

**Table 2:** Incidence and characteristics of recurrent tumor.

	Residual disease	Local recurrence	Synchronous lesion	Metachronous lesion
No. of recurrence, n (%)	7 (1.0)	6 (0.9)	7 (1.0)	31 (4.4)
Median duration of recurrence, month (range)	9 (3–12)	24 (18–60)	12 (12–12)	36 (15–84)
**Pathology of recurrence, n (%)**				
Adenoma	5 (0.7)	6 (0.9)	4 (0.6)	22 (3.2)
Low-grade dysplasia	4 (0.6)	5 (0.7)	4 (0.6)	19 (2.7)
High-grade dysplasia	1 (0.1)	1 (0.1)		3 (0.4)
Adenocarcinoma	2 (0.3)		3 (0.4)	9 (1.3)
Differentiated	2 (0.3)		2 (0.3)	6 (0.9)
Undifferentiated			1 (0.1)	3 (0.4)

Values are number of cases with percent in parentheses.

### Development of surveillance interval according to the risk group

**Figure 3:**
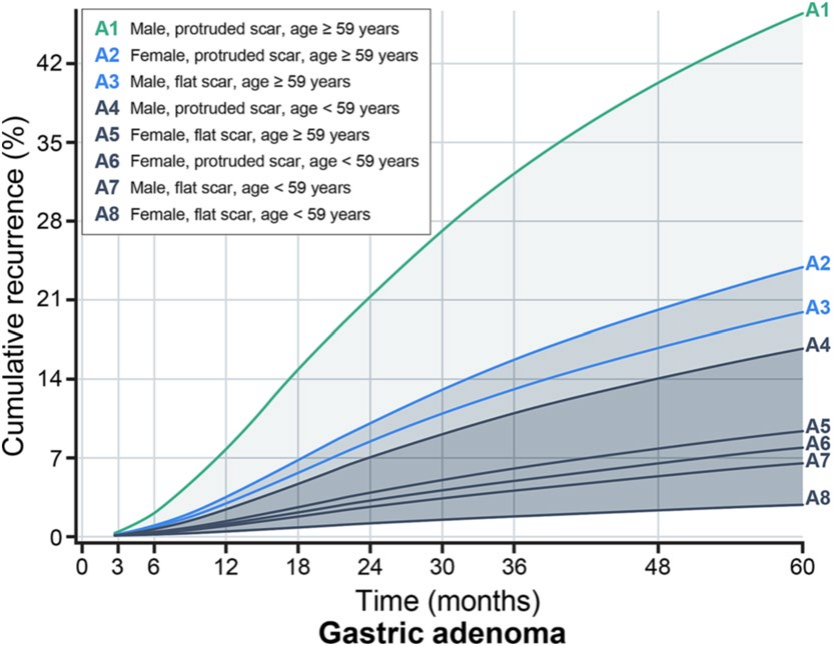
Cumulative density function plots for incidence of recurrence by risk factor categories in gastric adenoma after endoscopic submucosal dissection with curative resection. Current follow-up endoscopy after endoscopic submucosal dissection (ESD) was performed uniformly at 3, 6, 12, 18, 24 months and annually thereafter for all patients. The cumulative density of recurrence over time was estimated from the Cox proportional hazard model with interval censoring with identified risk factors. Using this estimated cumulative density plot, the surveillance time point when recurrence could be detected was assessed to not exceed the tolerance risk limit of 7%, and the cumulative risk between surveillance intervals on average was less than or equal to this limit. It is advised to have the risk-group specific follow-up schedule based on the risk group of a patient.

We investigated risk factors related to the recurrence in each patient using the Cox proportional hazard model and stepwise selection method as provided in Supplementary Table 1. As a result, the identified risk factors among all candidate risk factors were male sex (hazard ratio [HR], 2.60; 95% CI 1.096–6.172, *P* = 0.030), protruded scar at the 1st surveillance (HR, 3.18; 95% CI 1.601–6.300, *P* < 0.001), and age (HR, 1.05; 95% CI 1.023–1.086, *P* < 0.001). Age was converted into a binary variable to simply classification and interpretation, using a cutoff value of 59 years. On the basis of these results, we generated groups depending on these identified risk factors (Table 3). We investigated the cumulative density over months to find the appropriate surveillance interval for each risk group by conducting the Cox proportional regression model with interval censoring and a specified baseline hazard with smoothing by a cubic spline with 3 degrees of freedom. The development of this model enabled us to estimate the cumulative probability of developing recurrence over time for each risk group based on risk group-specific baseline hazards. The cumulative density function of recurrence plots by risk factor categories is shown in Fig. 3. Patients categorized as A1 (male, protruded scar at 3 months surveillance, age ≥ 59) showed the lowest survival rate (5-year recurrence free survival rate, 55.8%, 95% confidence interval, 39.1–79.8), which meant that they had the highest risks for recurrence (Table 3).

**Table 3:** Risk groups categorization according to the associated risk factor for recurrence after endoscopic submucosal dissection with curative resection gastric adenoma and five-year recurrence free survival rate.

Risk group	Risk factors	5-year recurrence free survival rate, % (95% CI)
A1	Male, protruded scar, age ≥ 59 years	55.8 (39.1–79.8)
A2	Female, protruded scar, age ≥ 59 years	77.2 (59.6–100)
A3	Male, flat scar, age ≥ 59 years	81.0 (74.9–87.7)
A4	Male, protruded scar, age < 59 years	84.0 (72.7–97.2)
A5	Female, flat scar, age ≥ 59 years	91.1 (84.9–97.7)
A6	Female, protruded scar, age < 59 years	92.6 (84.7–100)
A7	Male, flat scar, age < 59 years	93.9 (90.0–98.1)
A8	Female, flat scar, age < 59 years	97.3 (94.7–99.9)

CI, confidence interval.

In previous studies, all recurrence rates after ESD ranged between 1.8% and 32.4% (average curative resection rates: 89.9%, average recurrence rate: 8.1%)^9,19–23^. After checking whether the recurrence rates in our study were within this range, the tolerable risk limit of recurrence was set at 7.0%. The tolerable risk limit is defined as the maximum risk that a patient is willing to take for recurrence. The purpose of setting follow-up schedules and obeying them is to prevent a situation in which recurrence is identified too late for timely treatment. The risk of recurrence accumulates over time. The surveillance interval for each group was developed by using this recurrence limit for the generated risk groups. In the follow-up of patients in each group at the predicted time, patients were expected to yield a recurrence rate of less than 7.0% between the intervals. In the case of A1 (the highest risk group), the recurrence rate could be expected to fall below 7.0% at each surveillance visit only in case of surveillance at frequent intervals during the first 42 months. If there was no recurrence at first follow up after 3 months from ESD, the low-risk groups (A4, A5, A6, A7, and A8) did not need ESD surveillance for recurrence and were recommended to undergo endoscopy for cancer screening alone (Table 4). Thus, the recommended surveillance intervals can be applied according to the risk factors of the patient. We have simplified these results for clinical use. If the age factor is first classified, scar morphology and sex are sequentially judged; three categorizations can be made, and patients who do not fall under these categories are classified as “others.” A summary of the proposed optimal surveillance intervals according to the categorization is shown in Table 5.

**Table 4:** Suggested surveillance interval according to risk factor categorization in patients with gastric adenoma after endoscopic submucosal dissection with curative resection.

Group	Risk factors	Follow-up schedules after ESD																				Total visit^a^	Risk^b^ (%)
		3	6	9	12	15	18	21	24	27	30	33	36	39	42	45	48	51	54	57	60		
A1	Male	√			√		√		√			√			√				√			7	6.82
	Protruded scar																						
	Age ≥ 59 years																						
A2	Female	√					√					√							√			4	6.97
	Protruded scar																						
	Age ≥ 59 years																						
A3	Male	√						√							√						√	4	6.27
	Flat scar																						
	Age ≥ 59 years																						
A4	Male	√							√									√				3	6.96
	Protruded scar																						
	Age < 59 years																						
A5	Female	√															√					2	6.95
	Flat scar																						
	Age ≥ 59 years																						
A6	Female	√																	√			2	6.77
	Protruded scar																						
	Age < 59 years																						
A7	Male	√																			√	2	6.04
	Flat scar																						
	Age < 59 years																						
A8	Female	√																				1	–
	Flat scar																						
	Age < 59 years																						

ESD, endoscopic submucosal dissection.

^a^Predicted visit number by groups up to 60 months.

^b^Mean recurrence rate between visits.

**Table 5:** Suggested surveillance interval according to risk factor categorization in patients with gastric adenoma after endoscopic submucosal dissection with curative resection.

Risk factor 1	Risk factor 2	Risk factor 3	Suggested follow-up schedules after ESD	Total visit^a^
Age ≥ 59 years	Protruded scar	Male	12, 18, 24, 33, 42, and 54 months	6
		Female	18, 33, and 54 months	3
	Flat scar	Male	21, 42, and 60 months	3
Others			Routine endoscopic checkup is recommended^b^	2.5

The risk factors are determined after 3 months follow-up endoscopy in patients with endoscopic submucosal dissection. Suggested follow-up schedule is established when the patient has all three risk factors (risk factor 1, 2, and 3).

ESD, endoscopic submucosal dissection.

^a^This number excludes 3 months follow-up endoscopy (1st follow up).

^b^Biennial endoscopy is recommended if a protruded scar is noted at the 3-month follow-up endoscopy after ESD.

## Discussion

We conducted a study to provide optimal surveillance strategies by parameterizing diverse and group-specific recurrence risk factors to replace the uniform surveillance interval after ESD with curative resection for gastric adenoma. We found risk factors for recurrence after ESD to be distinguishable and categorized patients into eight groups with identified risk factors. Cumulative density function derived from the Cox proportional hazard model with interval censoring and tolerable risk limit of recurrence was used to find optimal surveillance intervals adapted for each risk group. Our findings suggest that surveillance intervals after ESD with curative resection should be different from current uniform intervals, and risk group-specific surveillance strategies may help reduce unnecessary examinations and increase required examinations. Although frequent surveillance endoscopic examinations detect recurrence early, surveillance is more important for patients with identified recurrence-related risk factors than those without.

A few studies have reported the risk factors for recurrence and outcomes after endoscopic resection, including ESD^9,24–27^. A study on adenoma alone found that the recurrence rate was 20.3% (27/133) (synchronous lesion: 8.3%, metachronous lesion: 12.0%) in a follow-up for more than two years after endoscopic resection (ESD: 94.0%, complete resection rate: 69.7%)^9^. In the same study, risk factors for recurrence were intestinal metaplasia and lesion size; however, they were not specific to curative resection cases. There are few studies on scar morphology. There are few studies on scar morphology. Arantes et al.^12^ reported that polypoid nodular scar be considered as a benign alteration, and no malignant recurrence was observed after curative resection. However, an interobserver disagreement may occur due to the ambiguity of determining scar morphology. Despite setting the definition of a protruded scar in this study, it is difficult to determine scar morphology accurately. Thus, further research regarding scar morphology is necessary.

In our study, the rate of recurrence at the ESD site (residual disease/local recurrence) differed significantly between patients with protruded scars and those with flat scars (8.9% vs. 1.3%, *P* = 0.002). Of the 11 cases of recurrence in patients with protruded scars, nine were adenomas. The mechanisms underlying protruded scar formation remain unclear, and it is difficult to explain the formation of protruded scars using risk factors for recurrence. Accurate pathologic evaluation may be more difficult for protruded scars than flat scars due to the sampling error associated with biopsies. Further studies are required to determine whether the changes reflect tumor formation or benign epithelial hyperplasia. At times, biopsies of ESD sites are conducted under the endoscopist’s discretion as well. However, in our study, biopsies of ESD sites were performed at all surveillance periods visits, allowing us to collect relatively accurate information for evaluation of the ESD site. Although further studies are required, our findings suggest that ESD site biopsy is an important component of surveillance endoscopy, especially in patients with protruded scars.

A few studies reported that the initial pathology, whether EGC or adenomas, had no effect on recurrence after endoscopic resection^9,21^. These studies indicated that selecting a high-risk group for adenomas is critical, and surveillance at the level of EGC is required; frequent endoscopic follow-up is needed for patients with high risk for gastric adenomas but can be reduced in the low-risk group. In our study, 68.1% of patients (475 patients) of the entire adenoma group are suggested to undergo endoscopic screening through the Korean National Cancer Screening Program.

We estimated the cumulative density function of recurrence from the Cox proportional hazard model with interval censoring and cubic spline baseline hazard for each risk group. The hazard rate was limited to the number of events per unit time and already determined by estimated models and assumptions. We facilitated the probability of occurrence of the incidence of recurrence in a given time interval. According to current data and literature, the recurrence limit was assumed at 7.0%. Based on this limit, we adjusted surveillance intervals to not exceed this limit; thus, patients following this surveillance schedule have a lower risk of recurrence between surveillance intervals.

There were some limitations to our study. First, this study was a retrospective and single-center study. Thus, selection bias might have occurred, and population validation is needed. While our study used 13-year data, a better surveillance schedule could be established if complemented by multicenter and multinational populations, as the selection of risk factors is significant. Second, the validation of tolerable risk limits used to determine the suggested surveillance intervals might have been required. Our study intervals were established based on recurrence rates in the literature and long-term data from our center; a difference in these values may result in a difference in the suggested surveillance interval. Third, the validation of tolerable risk limits used to determine the suggested surveillance intervals might have been required. In our study, the intervals were established based on recurrence rates in literature^9,19–23^ and the long-term data from our center because a difference in these values may result in a difference in the suggested surveillance interval. Fourth, we could not accurately evaluate the role of *H. pylori* infection in recurrence because this study has a retrospective design and lacks a time limit for the occurrence of reinfection after curative resection. The role of *H. pylori* infection and eradication in the recurrence requires further study. Fifth, although the percentage of patients with atrophic gastritis was 71.6% in our study, the proportion of uninfected patients with *H. pylori* were relatively high (47.0%). At our center, a rapid urease test and histopathologic evaluation are performed to check the status of *H. pylori* infection; however, this is associated with sampling error, which is insufficient to accurately confirm the infection status. Sixth, given the substantial duration of follow up in our study, we excluded many patients (n = 1029) due to short-term follow up only or the absence of 3-month endoscopic results, which may have caused selection bias. Seventh, although residual tumor/local recurrence and synchronous/metachronous gastric cancer are associated with different tumor characteristics, we did not include subdivisions based on recurrence type in the risk factor analysis, as we aimed to develop a simple surveillance strategy that relies on minimal risk factors for use in actual clinical practice. Eighth, it is difficult to clearly explain the recurrence at the ESD site (residual disease/local recurrence) after ESD with curative resection of the lesion. The current diagnosis was maintained in the pathologic review, but additional studies are needed to determine whether an evaluation is limited by cautery damage or by the occurrence of new lesions at the ESD site. Finally, although submucosal fibrosis did not affect recurrence, there was a difference in the baseline characteristics between the recurrence and non-recurrence groups, and we could not find the exact reason.

In conclusion, it is important to carry out surveillance endoscopy to detect recurrence after ESD with curative resection for gastric adenoma. Surveillance intervals could be changed depending on risk factors instead of a uniform surveillance endoscopic interval for all patients. The surveillance strategy should not be determined based on the final diagnosis alone. A surveillance interval strategy determined according to risk factors would enable physicians to detect recurrence in early stages, reduce the number of unnecessary surveillance examinations in patients and avoid additional costs, or encourage patients to undergo additional surveillance examinations for close monitoring and treatment. We believe this analysis is the first to provide a risk factor-based model of surveillance intervals after ESD by using the cumulative density of recurrence in the Cox proportional hazard model. Further research through multicenter and multination studies is required for validation, which will contribute to the development of an optimal surveillance strategy in patients with gastric adenoma after ESD.

## Supplementary Information

Supplementary Information.

## Data Availability

The datasets analyzed during the current study are available from the corresponding author on reasonable request.
