# Supplementary material for: Risk factor-based optimal endoscopic surveillance intervals after endoscopic submucosal dissection for gastric adenoma
Source: Sci Rep. 2021 Nov 1;11:21408. doi: 10.1038/s41598-021-00969-1 (PMC8560818; doi:10.1038/s41598-021-00969-1)
Supplement: Supplementary file 1 — Supplementary Information. [file 41598_2021_969_MOESM1_ESM.pdf]

Risk factor-based optimal endoscopic surveillance intervals after endoscopic submucosal dissection for gastric adenoma

Choong-Kyun Noh, Eunyoung Lee, Gil Ho Lee, Sun Gyo Lim, Kee Myung Lee, Jin Roh, Young Bae Kim, Bumhee Park, and Sung Jae Shin

Supplementary Figure

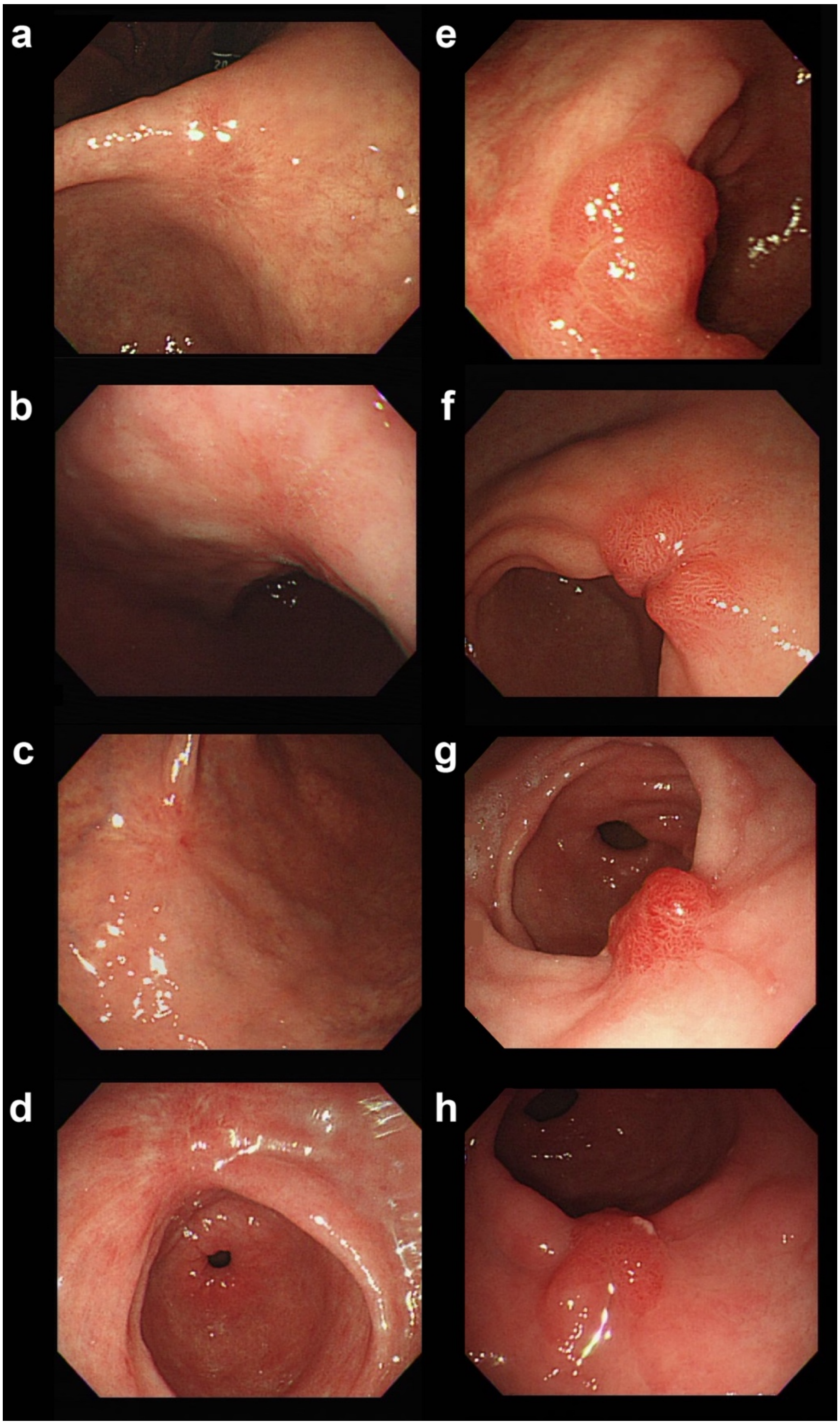

Supplement Figure 1. Various morphology of scar formation after endoscopic submucosal dissection (ESD). Clean scar was shown as flat, smooth, and whitish or reddish colored scarring change at the post-ESD scar site (a, b, c, and d). Protruded scar was characterized with polypoid or nodular shaped protuberant scarring formation located in the post-ESD site, which was accompanied with or without converging folds (e, f, g, and h).

Supplementary Table 1. Hazard ratios (95% confidence intervals) from univariate and multivariable Cox regression models and risk factors identified via Cox regression performed using a stepwise selection method for recurrence after endoscopic submucosal dissection with curative resection.

		Univariate analysis				Multivariable analysis				Stepwise selection			
Parameter		HR	95% CI		*P*-value	HR	95% CI		*P*-value	HR	95% CI		*P*-value
Sex	Male	2.89	1.232	6.798	0.015	2.17	0.955	4.954	0.064	2.60	1.096	6.172	0.030
	Female	ref.				ref.				ref.			
Number of lesion	Multiple	1.88	0.908	3.868	0.089	1.52	0.704	3.288	0.286				
	Single	ref.											
Atrophy gastritis	Yes	2.98	1.178	7.523	0.021	3.85	0.974	15.175	0.055				
	No					ref.							
H. pylori infection	Yes[a]	0.81	0.432	1.535	0.525	1.12	0.583	2.145	0.737				
	No	ref.				ref.							
Location	Upper third	ref.				ref.							
	Middle third	1.49	0.197	11.201	0.701	1.30	0.16	10.489	0.809				
	Lower third	1.81	0.247	13.241	0.560	1.42	0.179	11.298	0.738				
Gross morphology type	Depressed	0	0	.	0.984	0	0	.	0.988				
	Elevated/flat	ref.				ref.							
Ulceration	Yes	0.84	0.116	6.087	0.863	0.68	0.078	5.984	0.731				

	No	ref.				ref.							
Intestinal metaplasia	Yes	1.95	0.829	4.585	0.126	0.58	0.157	2.129	0.409				
	No	ref.				ref.							
Fibrosis during ESD	Yes	0.45	0.162	1.257	0.128	0.62	0.211	1.848	0.394				
	No	ref.				ref.							
Discrepancy	Upgrade	0.82	0.254	2.629	0.735	0.60	0.203	1.776	0.357				
	Downgrade and concordant	ref.				ref.							
Scar morphology at 1st follow-up	Protruded	3.48	1.779	6.824	0.0003	2.69	1.30	5.566	0.008	3.18	1.601	6.300	<0.001
	Flat	ref.				ref.				ref.			
Age		1.05	1.019	1.082	0.001	1.059	1.03	1.093	<0.001	1.05	1.023	1.086	<0.001
Lesion diameter, mm	≤10	ref.								ref.			
	10−20	1.12	0.487	2.586	0.786	1.039	0.44	2.442	0.930				
	20−30	1.28	0.503	3.276	0.601	1.17	0.432	3.139	0.763				
	>30	1.93	0.612	6.097	0.261	1.40	0.407	4.825	0.593				
Specimen area, mm^2		1.17	0.988	1.395	0.068	1.27	1.041	1.552	0.019				
Total procedure time, min		0.99	0.983	1.004	0.225	0.99	0.98	1.003	0.169				

HR, hazard ratio, CI, confidence interval, ref, reference, ESD, endoscopic submucosal dissection, *H. pylori, Helicobacter pylori*

[a]Included both current and previous infection (including eradicated status)
